# Virtual reconstruction of a provisional obturator using a mirroring technique in a patient with limited mouth opening

**DOI:** 10.1111/jopr.70006

**Published:** 2025-07-21

**Authors:** Min‐Ju Ji, Jung‐Jin Lee, Jae‐Min Seo, Yeon‐Hee Park

**Affiliations:** ^1^ Department of Prosthodontics, Institute of Oral Bio‐Science School of Dentistry, Jeonbuk National University and Research Institute of Clinical Medicine of Jeonbuk National University‐Biomedical Research Institute of Jeonbuk National University Hospital Jeonju Republic of Korea

**Keywords:** 3D printing, digital approach, digital impression, mirroring technique, provisional obturator, limited mouth opening

## Abstract

Capturing impressions for maxillary defect patients with trismus is challenging due to limited mouth opening and defect complexity. This study introduces an innovative digital technique that highlights virtual reconstruction through the integration of intraoral scanning and a mirroring approach. Using the intact maxillary arch as a reference, the defect area is digitally reconstructed to design a provisional obturator with a streamlined workflow. By eliminating the need for extensive laboratory procedures, this method significantly enhances the efficiency of prosthetic fabrication and simplifies the overall impression‐taking process.

For patients with large maxillofacial defects, extending the denture base into the defect area is essential not only to minimize excessive prosthesis movement but also to ensure functional stability and aesthetic rehabilitation. To achieve this, an accurate impression of the maxillary arch, including the defect site, must be obtained.^1^ However, as the size of the defect increases, capturing and removing a larger impression becomes challenging,^2^, ^3^ particularly in patients with trismus, a common complication following maxillectomy.^4^ Additionally, the overall fabrication process of a definitive obturator can be prolonged and complex, with studies reporting multiple visits over several months, especially in patients who have undergone radiation therapy.^5^

To address these challenges, the sectional impression technique has been commonly used.^6^, ^7^, ^8^ This approach involves modifying prefabricated trays and taking separate impressions for the normal and defect areas, which are then joined extraorally using interlocking indices or reassembled on casts.^6^, ^7^ While effective in some cases, these methods are time‐consuming, require multiple materials, and still present difficulties in removing large impressions from patients with limited mouth opening.

Intraoral scanners provide a promising alternative, offering easier access to the oral cavity due to their small scanning tips.^9^, ^10^ Moreover, recent clinical studies have demonstrated their effectiveness in fabricating maxillary obturator prostheses, particularly in patients with limited mouth opening, by minimizing the risk of impression material aspiration and simplifying the clinical workflow.^11^ Despite their advantages, capturing detailed data from defect sites remains a challenge. To overcome this, this article describes a novel technique that integrates intraoral scanning and virtual arch reconstruction to obtain patient data, combined with a conventional method for fabricating a provisional maxillofacial prosthesis in a trismus patient.

This technique was used to treat a patient with postoperative trismus following maxillectomy for a malignant neoplasm of the maxillary sinus. The tumor affected the pterygoid body, medial and lateral pterygoid plates, and associated muscles, necessitating an extensive maxillectomy, including removal of the right inferior turbinate, zygoma, and both medial and lateral pterygoid muscles (Figure 1). As a result, the patient developed severe trismus due to the significant loss of pterygoid musculature, resulting in an interincisal distance of 17 mm, classifying the patient as grade 2 according to the SOMA classification (Figure 2).^12^ The steps for fabricating the provisional prosthesis are described below.

**FIGURE 1:**
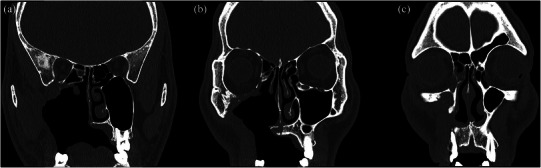
Coronal CT images showing the extent of a maxillary defect from the region of the second molar (a) to the canine region (c). Significant bone loss is observed, with involvement of the maxillary sinus and extension to the inferior orbital wall, highlighting the anatomical complexity and extensive nature of the defect.

**FIGURE 2:**
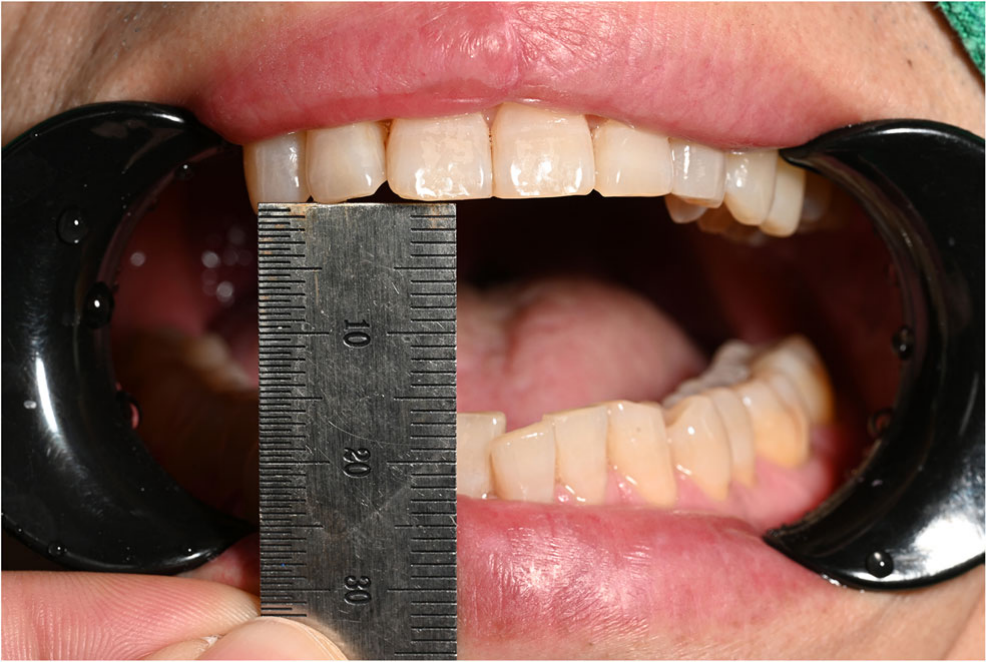
Maximum mouth opening–1.7 mm.

## TECHNIQUE

**FIGURE 3:**
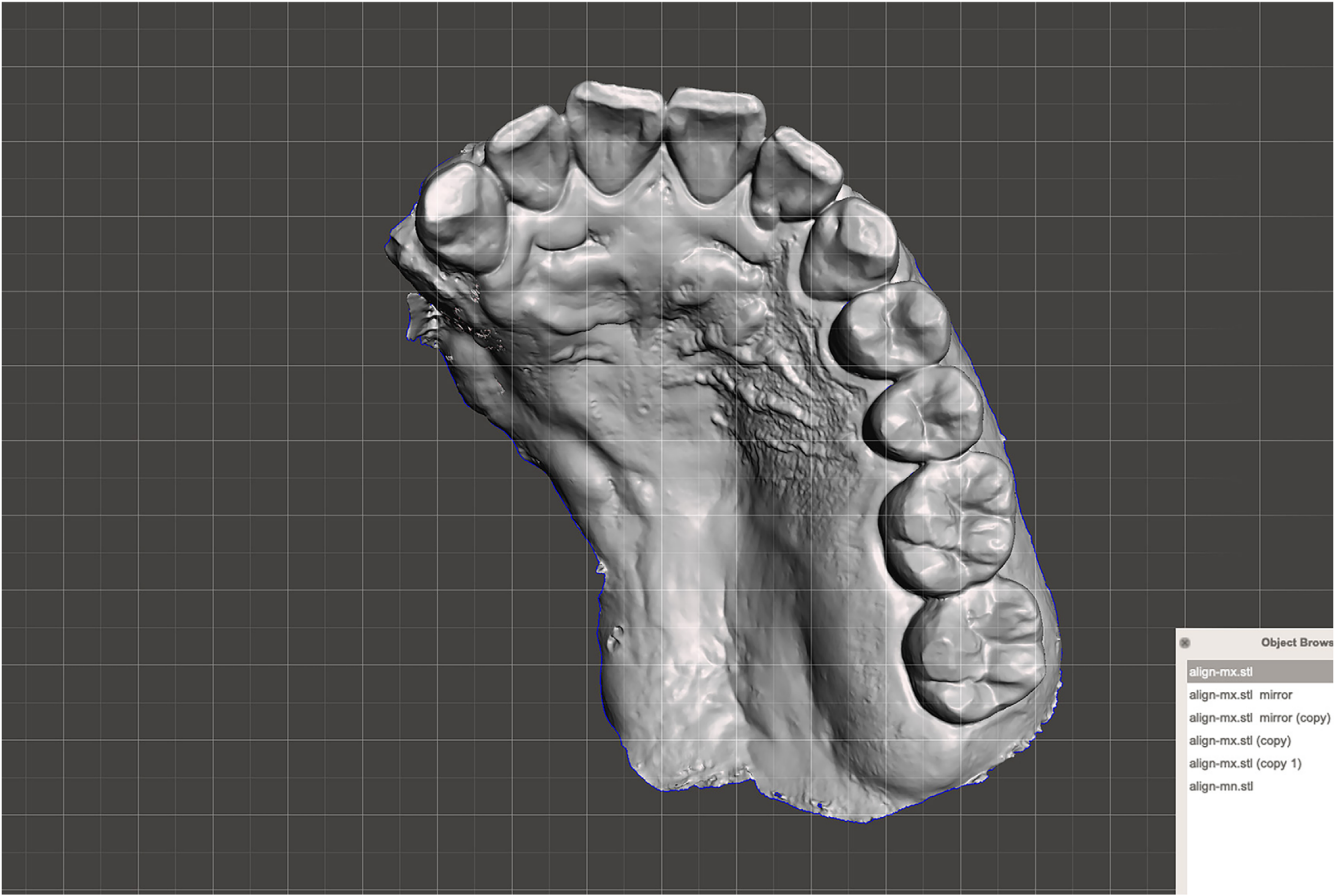
Scanned image of the patient's maxillary arch with an intraoral scanner. Defect extended from the #13 distal area to the mid‐palatine suture, beyond the hard and soft palate.

**FIGURE 4:**
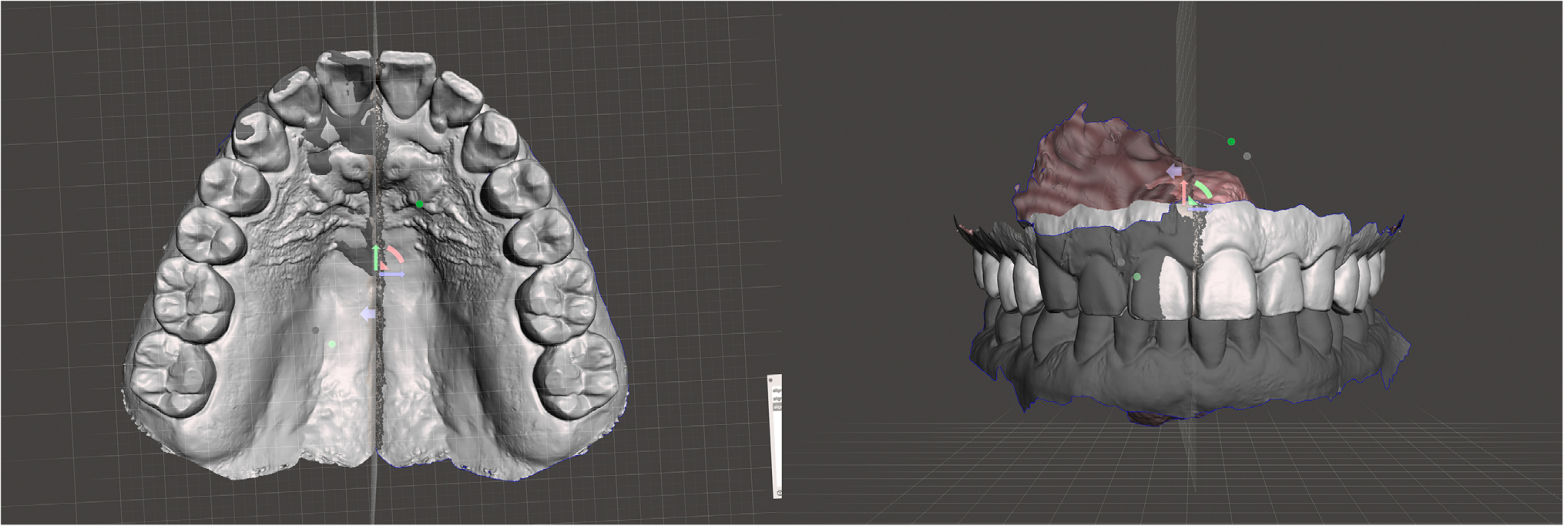
Data generated by mirroring the left maxilla across the median palatine suture.

**FIGURE 5:**
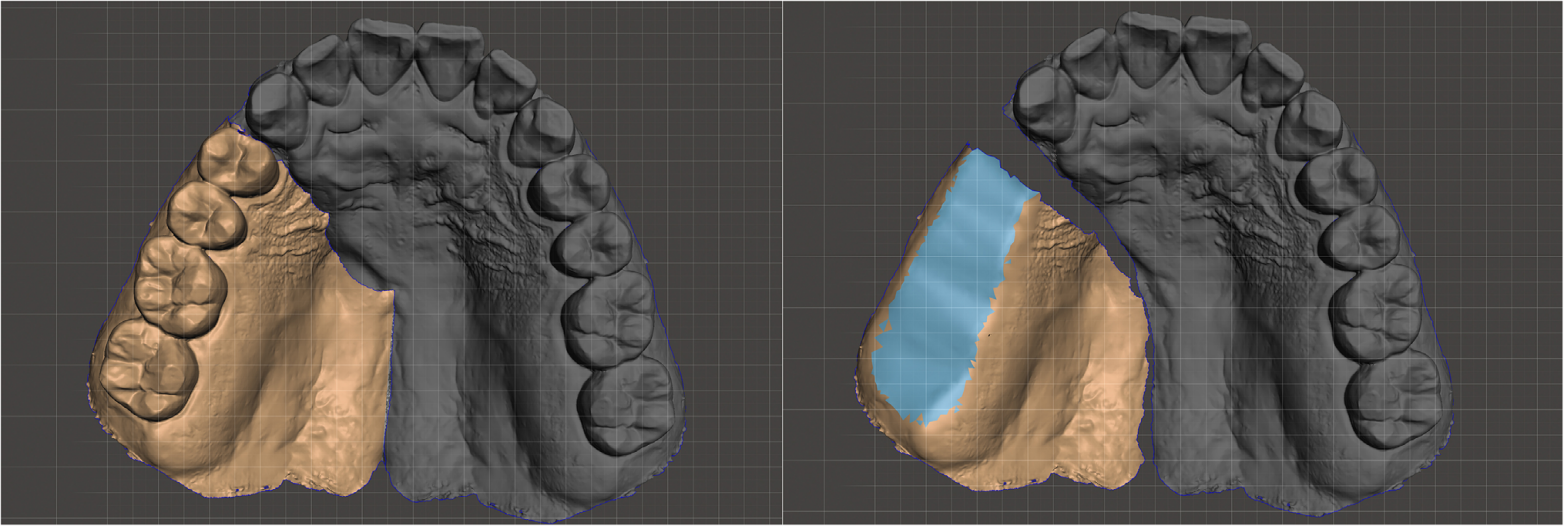
Overlapping areas and extraneous portions in the arch were trimmed.

**FIGURE 6:**
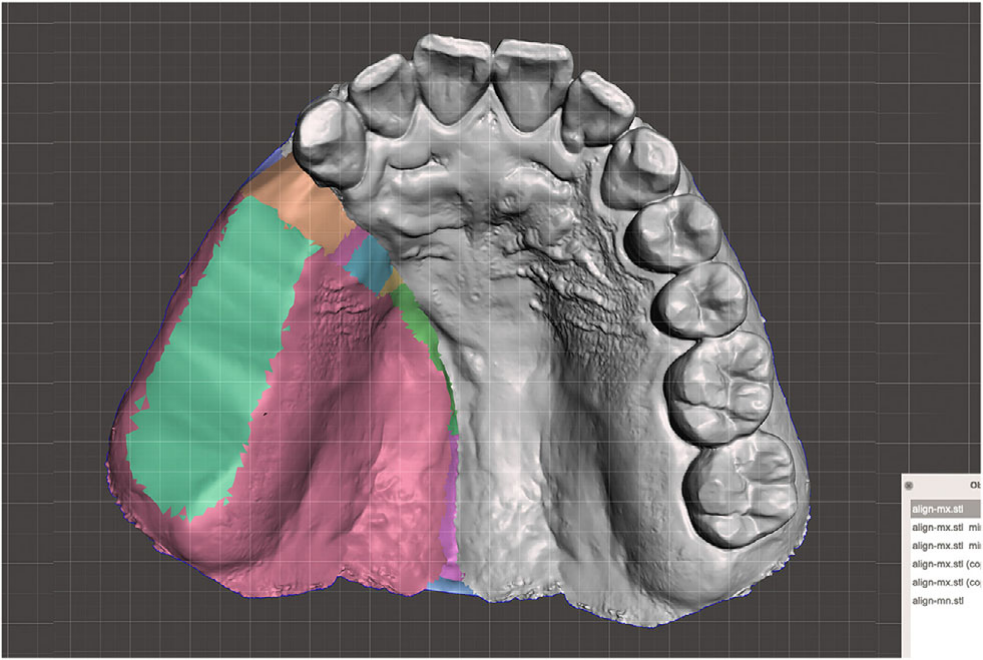
The final virtual maxillary arch.

**FIGURE 7:**
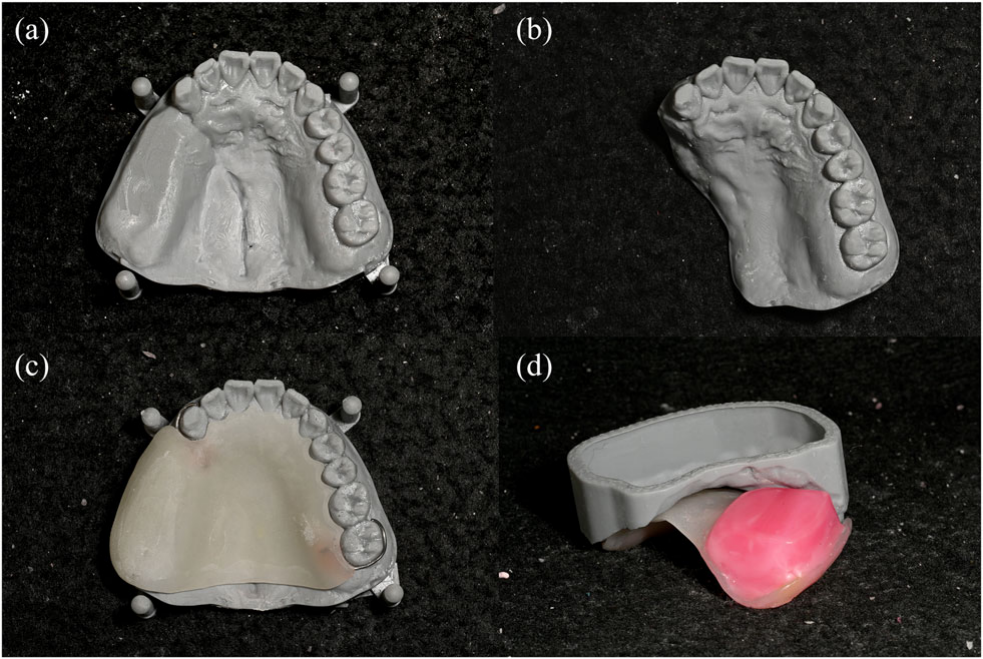
(a) 3D‐printed modified maxillary model. (b) 3D‐printed original maxillary model. (c) Resin base made on the modified maxillary model. (d) The resin base was adapted to the original maxillary model to make a modified tray by adding 1.5 mm of wax to fit the defect.

**FIGURE 8:**
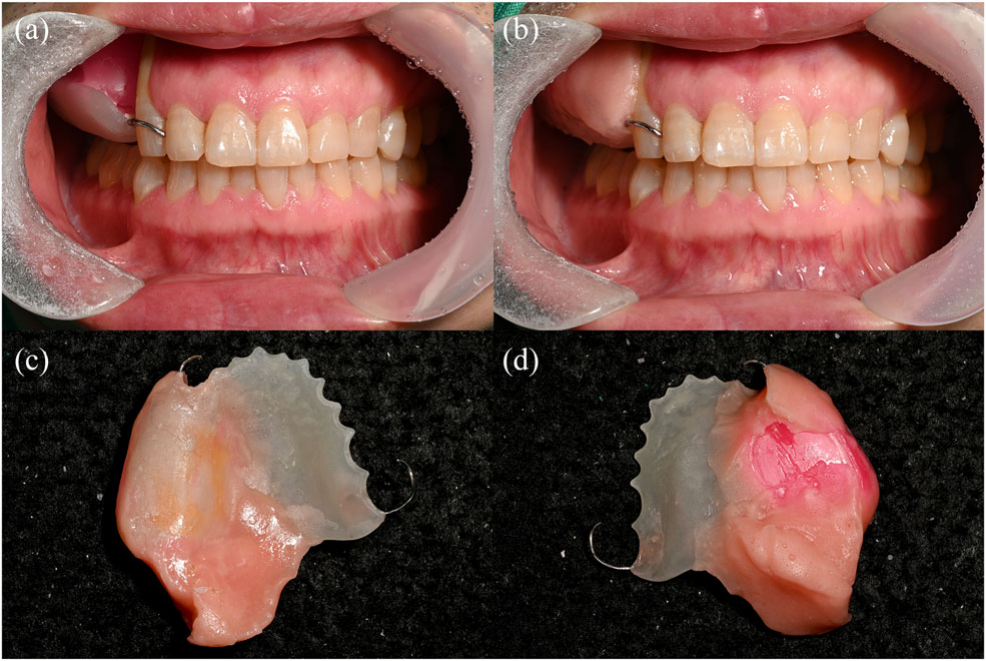
(a) Try‐in of the modified tray. (b) Final impression of the defect area was obtained using a wax denture and Coe‐soft. (c) Final impression. (d) The intaglio surface of the final impression.

**FIGURE 9:**
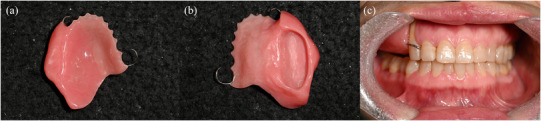
Provisional obturator. (a) External surface. (b) Internal surface. (c) Delivery of the provisional obturator.

1. Use an oral scanner (Medit i700, Medit Corp., Seoul, Korea) to acquire data from the residual maxillary teeth, alveolar ridge, hard and soft palate, and the accessible areas of the defect site (Figure 3).
2. Virtually reconstruct the right maxillary defect using the mirroring technique in universal CAD software (Meshmixer, Autodesk Inc., San Rafael, CA, USA). Copy and mirror the left half of the arch to the right, using the mid‐palatine suture as the reference line (Figure 4).
3. Refine the mirrored data by removing overlapping regions, such as the right maxillary central incisor to the canine, and eliminating extraneous portions, including crowns from the first premolar to the second molar on the mirrored side (Figure 5).
4. Integrate the mirrored maxillary arch with the original arch using the “Bridge” and “Inspector” functions in Meshmixer. Export an STL file of the modified maxillary arch (Figure 6).
5. 3D print the modified and original maxillary models using NextDent Model 2.0 (NextDent, Soesterberg, Netherlands) and NextDent 5100 (3D Systems, Rock Hill, SC, USA), respectively (Figure 7a, b). Use the modified maxillary model to fabricate a resin base for the modified tray (Figure 7c). Fit the resin base onto the original maxillary model to confirm compatibility. Add approximately 1.5 mm of wax on the resin base to ensure it fits the defect site on the original maxillary model, optimizing tray adaptation while maintaining ease of insertion and removal within the patient's limited mouth opening (Figure 7d). Confirm the intraoral fit of the modified tray.
6. Insert the modified tray into the patient's mouth and confirm its removability. An impression of the defect area is made using Coe‐Soft (GC America Inc., Alsip, IL, USA) and the modified tray, ensuring that the impression covered a range that allowed easy insertion and removal (Figure 8).
7. Polymerize the impression using SR Ivocap (Ivoclar Vivadent, Schaan, Liechtenstein) to fabricate the provisional prosthesis (Figure 9).

## DISCUSSION

In dentistry, digital technology is primarily utilized to obtain intraoral data and fabricate prostheses. Intraoral scanners, in particular, are advantageous for impression making in patients with trismus, as their small tip size allows easier access compared to conventional impression trays.^8^, ^9^ However, in patients with both maxillofacial defects and trismus, even if the scanner tip can be inserted into the oral cavity, accurately capturing the defect area remains challenging. Since precise defect information is essential for prosthesis fabrication, an alternative approach is required.

One of the key advantages of digital techniques is the ability to freely modify and manipulate data in a virtual environment. Functions such as copy‐and‐paste, redo, and undo allow for efficient modifications, and digital files can be saved in multiple iterations to accommodate various design refinements. Mirroring techniques can also be applied to create symmetrical structures and are commonly used to replicate the shape of existing teeth for final prosthesis fabrication, particularly in aesthetically demanding regions such as the maxillary anterior area. In this technique, the concept of mirroring was extended by reflecting the intact maxillary arch onto the defect site, enabling the virtual reconstruction of the missing structure. This reconstructed model served not only as a reference for design, but also as the foundation for creating a customized impression tray tailored to the patient's limited mouth opening—positioning the mirroring‐based tray fabrication as the central component of this approach.

Obtaining an impression in maxillofacial defect patients, especially those with limited mouth opening, is highly challenging. As the defect size increases, the impression also becomes larger, making the tray insertion and impression removal even more difficult due to restricted mouth opening. In this technique, a mirroring method was used to reconstruct a virtual maxillary arch, which served as the foundation for fabricating a modified impression tray. The modified tray was designed by considering the vertical morphology of the defect and the patient's mouth opening capacity, with wax selectively added to the defect side to achieve an appropriate impression size.

Additionally, the modified tray was engineered to ensure adequate sealing at the defect site while allowing for controlled impression making within the removable range permitted by the patient's mouth opening. The final impression obtained through this process was then used to fabricate a provisional maxillary obturator using conventional techniques.

This method can be effectively applied to maxillofacial defect patients with limited mouth opening, maximizing the advantages of digital editing to overcome challenges in impression making and model fabrication. Compared to conventional sectional impression methods, which often require multiple impression steps, tray modifications, and complex reassembly procedures, this technique offers a more streamlined and time‐efficient workflow. It reduces the need for extensive laboratory work and minimizes patient discomfort during impression taking, while still allowing for adequate border extension through the use of a soft relining material. However, this approach relies on the presence of sufficient remaining anatomical structures to guide virtual reconstruction, and may be less suitable for cases involving bilateral defects or highly irregular anatomy where precise mirroring is not feasible.

Additionally, as the virtually reconstructed maxillary arch may not perfectly correspond to the actual patient anatomy, further modifications should be incorporated during final impression‐taking to ensure optimal accuracy.

## SUMMARY

This technique utilizes intraoral scanning and mirroring to efficiently fabricate provisional prostheses for patients with maxillary defects and limited mouth opening. By digitally reconstructing the defect area using the intact maxillary arch as a reference, it eliminates the need for complex laboratory procedures or extensive tray modifications. Integrating digital tools with conventional methods enhances efficiency, accuracy, and accessibility in treating maxillofacial defect patients with trismus.
